# Mitigation of *Paeoniae Radix* Alba extracts on H_2_O_2_-induced oxidative damage in HepG2 cells and hyperglycemia in zebrafish, and identification of phytochemical constituents

**DOI:** 10.3389/fnut.2023.1135759

**Published:** 2023-02-23

**Authors:** Lu Zhang, Mei Deng, Si-yu Wang, Qiao Ding, Jia-hui Liu, Xing Xie, Yun-hong Huang, Zong-cai Tu

**Affiliations:** ^1^National R&D Center for Freshwater Fish Processing, College of Life Science, Jiangxi Normal University, Nanchang, Jiangxi, China; ^2^Jiangxi Deshang Pharmaceutical Co., Ltd., Yichun, Jiangxi, China; ^3^State Key Laboratory of Food Science and Technology, Nanchang University, Nanchang, Jiangxi, China

**Keywords:** *Paeoniae Radix* Alba, hyperglycemia, oxidative damage, peripheral nerve protection, phytochemical identification

## Abstract

*Paeoniae Radix* Alba (PRA), as a Traditional Chinese Medicine, is widely used in Chinese cuisine due to high health-benefits and nutrition, but the effect of different polarity of solvents on the extraction of antioxidant and hypoglycemic constituents, as well as the major active compounds remain unclear. In this research, 40, 70, and 95% ethanol were firstly applied to extract the polyphenols from PRA, the extraction yields, total phenolics, and total flavonoids content, free radical scavenging ability, α-glucosidase inhibition ability, and anti-glycation ability of extracts were evaluated spectroscopically. The oxidative damage protection, hypoglycemic activity, and alleviation on peripheral nerve damage were evaluated by H_2_O_2_-induced HepG2 cells and hyperglycemic zebrafish models. UPLC-QTOF-MS/MS was used to identify the major chemical constituents. The results showed that 40, 70, and 95% ethanol exhibited insignificant difference on the extraction of phenolics and flavonoids from PRA. All extracts showed promising DPPH⋅ and ABTS⋅^+^ scavenging ability, α-glucosidase inhibition and anti-glycation ability. In addition, PRA extracts could restore the survival rate of HepG2 cells induced by H_2_O_2_, and alleviate the oxidative stress by reducing the content of MDA and increasing the levels of SOD, CAT, and GSH-Px. The 70% ethanol extract could also mitigate the blood glucose level and peripheral motor nerve damage of hyperglycemic zebrafish. Thirty-five compounds were identified from 70% ethanol extract, gallotannins, gallic acid and its derivatives, and paeoniflorin and its derivatives were the dominant bioactive compounds. Above results could provide important information for the value-added application of PRA in functional food and medicinal industry.

## 1. Introduction

Diabetes mellitus (DM) is a chronic metabolic disease characterized by continuous hyperglycemia due to either insulin deficiency or insulin resistance, along with metabolic disturbance of carbohydrate, fat, and protein. According to the International Diabetes Federation, the global diabetes prevalence in 2021 reached 10.5% (536.6 million people), and was expected to be 12.2% in 2045 (783.2 million people) (1). The economic growth, urbanization, reduction of physical activity, and dietary habits are the main reasons for the rising of DM (2). Prolonged hyperglycemia will damage multiple vital organs tissues (eyes, kidneys, heart, nerves, and blood vessels), resulting in many complications, such as neuropathy, nephropathy, retinopathy, and hypertension (3). Currently, hypoglycemic drugs (biguanides, thiazolidinediones, α-glucosidase inhibitors, etc.) and injection of insulin are the major treatment of DM (4). However, some of these approaches cannot effectively avoid the occurrence of complications and may result in many side effects, such as weight gain, hypoglycemia, gastrointestinal disturbances, flatulence, diarrhea, abdominal pain, and others (5). Therefore, the development of new natural anti-diabetic resource with low side effects has great application prospect.

Furthermore, strong evidences showed that oxidative stress plays an important role during the development of DM and its complications through activating the metabolic pathways of glycolytic pathway, hexosamine pathway, and polyol pathway (6). Oxidative stress was activated by the over production of reactive oxygen species (ROS) or the reduction of antioxidants, or both. Under the hyperglycemia conditions, the excessively produced ROS destroyed the steady state of reduction-oxidation (redox), resulting in oxidative damages of biomolecules (proteins, lipid, and DNA), formation of advanced glycation end products (AGEs), and dysfunction of β-cell and endothelial cells (7, 8). Moreover, oxidative stress was reported to stimulate damage to nerve cell with exhaustion of cell antioxidants and generation of pro-inflammatory signals in the diabetic neuropathy (9). Based on the close relationship between oxidative stress, DM and its complications, seeking of new therapeutic approaches focusing on oxidative stress are of great significance.

Natural extracts had attracted great attention because of their low side effects and promising protective effect on diabetes, inflammation, cancer, neurodegenerative conditions, etc. *Paeoniae Radix* Alba (PRA, Bai Shao in China) is the dried root of *Paeonia lactiflora* Pall, which has been used as traditional medicine for centuries in attenuating liver diseases, regulating menstruation and rheumatism, nourishing blood, and relieving pain (10). It is also widely used in Chinese food due to its nutrition and healthcare function, especially in various stews and soups, such as stewed PRA with pig’s feet, stewed PRA with pigeon, oyster-PRA soup, et al. Recently, PRA extracts were found to exhibit diverse pharmacological activities, such as anticancer, antioxidant, immunomodulation, anti-inflammatory, and anti-diabetic properties due to its rich bioactive compounds, including monoterpene glycosides, tannins, volatile oils, polysaccharides, etc. (10, 11). Juan et al. (11) indicated that PRA extracts exhibited anti-diabetic functions by stimulating glucose uptake, inhibiting glucose absorption and gluconeogenesis transcription. The total glucosides of PRA showed a protective effect on renal function in diabetic nephropathy by increasing T-AOC, SOD, and CAT activities, and reducing the level of MDA (12). However, systematic research on the antioxidant and hypoglycemic activities of different PRA extracts *in vitro* and *in vivo*, and the identification of primary active compounds are still needed further study, which will provide more sufficient basis for the scientific application of PRA.

In this article, PRA was extracted with 40, 70, and 95% ethanol. The antioxidant and hypoglycemic activities *in vitro* were evaluated by DPPH⋅ and ABTS⋅^+^ scavenging ability, α-glucosidase inhibition, and bovine serum albumin (BSA) glycosylation assays. Furthermore, to further evaluate the antioxidant capacity, the oxidative protection and influence on the activities of antioxidant enzymes were measured with HepG2 oxidative injury models induced by H_2_O_2_. *In vivo* hypoglycemic and peripheral motor nerve protective ability were evaluated with hyperglycemic zebrafish model. Finally, the major chemical composition was preliminarily identified by high performance liquid chromatography-high resolution mass spectrometry (HPLC-QTOF-MS/MS), which provided theoretical basis for comprehensive utilization of PRA.

## 2. Materials and methods

### 2.1. Materials and chemicals

Dried PRA was purchased from Anqing Chunyuan Pharmacy (Anhui, China). Pioglitazone hydrochloride tablets were bought from Deyuan Pharmaceutical Co., Ltd. (Jiangsu, China). Glucose, gallic acid, quercetin, and ethanol were from Aladdin Biotechnology Technology (Shanghai, China). Acarbose, 1-diphenyl-2-picrylhydrazyl (DPPH⋅), 4′-nitrophenyl-beta-D-glucopyranoside (pNPG), 2,2-azinobis-(3-ethylbenzothiazoline-6-sulfonic acid) (ABTS⋅^+^), and α-glucosidase were purchased from Sigma-Aldrich (St. Louis, MO, USA). HepG2 cell and Dulbecco’s modified Eagle’s medium (DMEM) with high sugar medium were from Beina Biology (Beijing, China). Amino guanidine was from Bio-Rad Laboratories Ltd. (Shanghai, China). Sitagliptin was bought from Macklin (Shanghai, China). All other chemicals were analytical grade and purchased from Sinopharm Chemical Reagent Co., Ltd. (Shanghai, China).

### 2.2. Sample preparation

The PRA powder (5.0 g) was homogenized in 40, 70, and 95% ethanol aqueous solution, respectively (1:20, w/v). Then the mixtures were treated with KQ-300DE Ultrasonic cleaner (Kun Shan Ultrasonic Instruments Co., Ltd., China) at 50°C, 400 W for 1 h. After centrifugation at 7,000 rpm for 5 min, the supernatants were collected, the residues were re-extracted according to the same method. Finally, the supernatants were combined and evaporated, the volumes were adjusted to 100 ml with extraction solvent to obtain the extracts for further analysis. E40, E70, and E95 was used to refer to the extracts prepared with 40, 70, and 95% ethanol aqueous solution, respectively. Meanwhile, 2.0 ml of extracts were taken out and lyophilized with LGJ-1D-80 Freeze Drier (Beijing Yatai Kelong Instrument Technology Co., Ltd., China) to calculate the concentration of extract, the yield of extract was calculated as follows:


(1) $$\text{Yield}\left(\%\right)=\frac{\text{conc}\,\mathrm{entration}\,\mathrm{of}\,\mathrm{extracts}\,\left(\mathrm{mg}/m\,\text{l}\right)}{\mathrm{concentration}\,\mathrm{of}\,\mathrm{dry}\,\mathrm{sample}\,\left(\mathrm{mg}/m\,\text{l}\right)}\times 100\%$$

### 2.3. Determination of total phenolics and total flavonoids content

#### 2.3.1. Total phenolics content

The total phenolics content (TPC) was evaluated using Folin–Ciocalteu method as reported (13). The absorbance at 765 nm was measured using Synergy H1 microplate reader (Biotek Vermont, USA). The calibration curve (*Y* = 0.0058x + 0.0511, *R*^2^ = 1.00) was constructed with gallic acid as standard to calculate the TPC in PRA extracts. All results were expressed as μg of gallic acid equivalent (GAE) per milliliter of extraction solution (μg GAE/ml).

#### 2.3.2. Total flavonoids content

The total flavonoids content (TFC) was measured using AlCl_3_-NaOH-NaNO_3_ method according to the report of Zhang et al. (14). Absorbance at 430 nm was measured using Synergy H1 microplate reader (Biotek Vermont, USA). The calibration curve (*Y* = 0.0016x + 0.056, *R*^2^ = 0.9985) was constructed with quercetin as standard to calculate the TFC in PRA extracts. All results were expressed as μg of quercetin equivalent (QuE) per milliliter of extraction solution (μg QuE/ml).

### 2.4. Determination of radical scavenging ability

The DPPH⋅ and ABTS⋅^+^ scavenging activity of samples were analyzed according to the method reported by Jia et al. (15). The PRA extracts at different concentrations (50 μl) and DPPH⋅ or ABTS⋅^+^ working solution (150 μl) were mixed in 96-well microplate, then the mixtures were incubated for 30 min or 6 min at room temperature in darkness. The absorbance at 510 or 734 nm was measured using a Synergy H1 microplate reader (Biotek Vermont, USA). Quercetin and ethanol were used as positive and negative control, respectively. The radical scavenging activity was calculated as follows:


(2) $$\mathrm{Scavenging}\mathrm{activity}\left(\%\right)=\frac{\left(A_{\text{c}}-{\text{A}}_{\text{b}}\right)-\left({\text{A}}_{\text{i}}-{\text{A}}_{\text{ib}}\right)}{{\text{A}}_{\text{c}}-{\text{A}}_{\text{b}}}\times 100\%$$

where A_c_ was the absorbance of control group; A_b_ was the absorbance of blank group without samples and free radical solution; A_i_ was the absorbance of sample group, and A_ib_ was the absorbance of sample blank group without free radical solution only. The IC_50_ values were calculated based on the curves of sample concentration vs. scavenging activity by Origin 2019 (OriginLab Co., Ltd., USA).

### 2.5. Protective effect against oxidative damage in H_2_O_2_-induced HepG2 cells

#### 2.5.1. Cell culture and cells viability assay

The HepG2 cells were cultured in DMEM medium containing 10% fetal bovine serum (FBS), 100 units/ml penicillin and 100 μg/ml streptomycin. To determine the toxicity of PRA extracts and H_2_O_2_, the viability of HepG2 cells was measured by CCK-8 assay (16). Briefly, the HepG2 cells (8.0 × 10^3^ cells/well) were incubated in 96-well plates at 37°C for 24 h in an MCO-18AC incubator (Panasonic, Japan) containing 5% CO_2_, followed by the addition of PRA extracts (25, 50, 100, 200, 400, and 800 μg/ml) for 24 h cultivation or H_2_O_2_ (400, 500, 600, and 700 μM) for 6 h cultivation. Then, CCK-8 solution (10 μl/well) was added for another 2 h of incubation, the absorbance of each well at 450 nm was determined with SMR60047 microplate reader (USCNK, China).

#### 2.5.2. Cytoprotective effect on H_2_O_2_-induced cell damage

After incubation in a 96-well plate (8.0 × 10^3^ cells/well) for 24 h, HepG2 cells were pre-treated with PRA extracts (37.5, 75, and 150 μg/ml) for 24 h, followed by incubation with H_2_O_2_ (600 μM) for 6 h. Cells only treated with H_2_O_2_ were used as model group (oxidative stress). The CCK-8 assay kit was used to determine the cytoprotective effects of PRA extracts against H_2_O_2_-induced oxidative damage.

#### 2.5.3. Measurement of antioxidant enzymes

After the establishment of oxidative damage in H_2_O_2_-induced HepG2 cells as described in section “2.5.2. Cytoprotective effect on H_2_O_2_-induced cell damage,” cells were lysed and centrifuged, the supernatant was used to measure antioxidant enzyme activities. The antioxidant activities of PRA extracts on HepG2 cells were detected using SOD, MDA, CAT, and GSH-Px assay kits (Jiancheng Bioengineering Institute, Nanjing, China) according to the instructions. The protein content of HepG2 cells was detected by the BCA assay kit (Jiancheng Bioengineering Institute, Nanjing, China).

### 2.6. Inhibitory activity on α-glucosidase

The α-glucosidase inhibitory activity was detected as previously reported (13). Briefly, 50 μl PRA extraction solutions at different concentrations and 50 μl phosphate buffered saline (PBS) were incubated with 100 μl α-glucosidase solution (pH 6.9, 0.2 U/ml, in 0.1 M PBS) in 96-well plates at 25°C for 10 min. Then, 50 μl, 5 mM pNPG solution was added to each well. After 10 min of incubation at 25°C, Na_2_CO_3_ solution (100 μl, 0.2 M) was mixed to terminate the reaction, and absorbance at 405 nm was measured using a Synergy H1 microplate reader (Biotek Vermont, USA). Acarbose was used as positive control, the IC_50_ values were used to reflect the inhibition ability.

### 2.7. Inhibition on bovine serum albumin-fructose glycosylation

The glycosylation inhibition was measured with BSA-fructose glycosylation model (17). The BSA solution (1.0 ml, 625 mM), fructose solution (1.0 ml, 20 mg/ml), and PRA extracts (100 μl, 1.0 mg/ml) were mixed and reacted at 55°C for 24 h. All sample solutions were 10-fold dilution prior to the measurement of fluorescence intensity at an excitation and emission wavelength of 350 and 425 nm, respectively. Hitachi F-7000 fluorescence spectrometer (Tokyo, Japan) was used to record the data, amino guanidine was taken as positive control. The inhibition rate of PRA extracts on BSA glycosylation was calculated as following:


(3) $$\mathrm{Inhibition}\mathrm{rate}\left(\%\right)=\frac{\left({\text{FI}}_{\text{c}}-{\text{FI}}_{\text{b}}\right)-\left({\text{FI}}_{\text{s}}-{\text{FI}}_{\text{nb}}\right)}{{\text{FI}}_{\text{c}}-{\text{FI}}_{\text{b}}}\times 100\%$$

where FI_s_, FI_c_, FI_b_, and FI_nb_ were the fluorescence intensities of PRA extracts group, control group (without PRA extracts), blank group (without fructose and PRA extracts) and sample blank group (without fructose), respectively.

### 2.8. *In vivo* hypoglycemic effects and protection on nerve injury

#### 2.8.1. Determination of maximum detection concentration

Wild type AB zebrafish (390) aged 5 days were propagated and breed by Hunter Biotechnology Co., Ltd. accredited by the Association for Assessment and Accreditation of Laboratory Animal Care (AAALAC) International. They were randomly selected in a beaker containing 25 ml water with 30 in each group, and freely feed at 28°C under light/dark cycle conditions. The hyperglycemic zebrafish model was established with high-sugar and high-fat diet. Then, the E70 PRA extracts at 62.5, 125, 250, 500, 1,000, and 2,000 μg/ml were added into the water, respectively. During the experiment period, the numbers of zebrafish deaths in each group were calculated and removed in time. After 2 days of continuous treatment for 7.5 h/day, maximum detection concentration (MTC) of zebrafish with hyperglycemia model was determined. All procedures were approved by the Institutional Animal Care and Use Committee at Hunter Biotechnology, Inc. [approval number: IACUC-2020-2574-01, use license number: SYXK (zhe) 2022-0004]. The feeding and management were accredited by the Association for Assessment and Accreditation of Laboratory Animal Care (AAALAC) International (No. 001458).

#### 2.8.2. Evaluation of hypoglycemic effects

After the determination of MTC, the hyperglycemic zebrafish model was established as described in section “2.8.1. Determination of maximum detection concentration.” The zebrafish without any treatment was taken as normal group. The zebrafish treated with 500, 1,000, and 2,000 μg/ml of E70 PRA extract were taken as treatment groups, while the zebrafish treated with 18 μg/ml pioglitazone (PGTZ) was used as positive control. The blood glucose level (G) was determined with blood glucose meter, the hypoglycemic effect was calculated as follows:


(4) $$\mathrm{Hypoglycemic}\mathrm{effect}\left(\%\right)=\frac{\text{G}\,\left(\text{model}\right)-G\,\left(\mathrm{sample}\right)}{G\,\left(\mathrm{model}\right)}\times 100\%$$

#### 2.8.3. Evaluation of peripheral motor nerve protection

The transgenic motor neuron green fluorescent strain zebrafish (NBT, 270) aged 5 days (dpf) were randomly selected and feed in a beaker containing 50 ml water (30 zebrafish in each group). The hyperglycemic zebrafish model was established as described in section “2.8.1. Determination of maximum detection concentration,” following by the treatment with 1,000, 1,500, and 2,000 μg/ml E70 PRA extract at 28°C for 7.5 h/day. After 2 days treatment, 10 zebrafish in each group were randomly selected and photographed with AZ100 fluorescence microscope (Nikon, Japan). The fluorescence intensity (S) of peripheral motor nerve in the area of two segments above the ventral pores of zebrafish was analyzed by NIS-Elements D 3.20. Sitagliptin (STGP, 350 μg/ml) was used as positive control, the protective effect on peripheral nerve was calculated as follows:


(5) $$\mathrm{Peripheral}\mathrm{nerve}\mathrm{protective}\mathrm{effect}\left(\%\right)=\frac{\text{S}\,\left(\text{model}\right)-S\,\left(\mathrm{sample}\right)}{S\,\left(\mathrm{model}\right)}\times 100\%$$

### 2.9. Identification of phytochemical profiling

Isolation and identification of the major phytochemical constituents in the E70 of PRA was carried out on a UPLC LC30 SYSTEM (Shimadzu, Japan) coupled to a Hybrid Quadrupole-TOF Mass Spectrometer Triple TOF 5600^+^ system (AB SCIEX, USA). Compounds were separated on a YMC C18 column (4.6 × 250 mm, 5 μm, GL Science, Japan) at a flow rate of 0.8 ml/min. Acetonitrile and 0.1% formic acids were used as mobile phases A and B, respectively. The optimized gradient elution program was: 0 min, 5% A; 6 min, 9% A; 7 min, 18% A; and 30 min, 40% A. All samples were filtered through a 0.22 μm membrane prior to injection (10 μl). The MS and MS/MS data were collected under negative ion mode in the scan range of m/z 100–1,500 and 50–1,500, respectively, with electrospray ionization (ESI) resource. The MS and MS/MS data were processed with software PeakView 1.2.

### 2.10. Statistical analysis

The experiments *in vitro* were repeated for three times. The results were expressed as mean standard deviation. One-way analysis of variance (ANOVA) with Tukey’s *b* test was carried out by SPSS 22.0 (Armonk, NY, USA) to assess the statistical significance difference among data, *P* < 0.05 refers significant difference.

## 3. Results

### 3.1. Comparison of yield, TPC, and TFC

Phenolics and flavonoids were the main active components of PRA (18), their extraction efficacy will impact its further processing efficacy and the bio-activity of extracts, thus, influence of different polarity of solvents on the extraction of phenolics and flavonoids from PRA were evaluated. As indicated in Table 1, 70% ethanol showed the highest extraction yield, with the percentage rate of 18.1%, followed by 95% ethanol, and 40% ethanol. While, the TPC in the extraction solutions were determined to be 281.94 ± 3.36, 276.82 ± 8.11, and 273.20 ± 9.55 μg GAE/ml, respectively, for 40, 70, and 95% ethanol solution, insignificant difference was observed (*P* > 0.05). Meanwhile, different ethanol concentrations also have insignificant influence on the concentration of total flavonoids, the values were 37.92 ± 5.64, 36.67 ± 6.91, and 40.63 ± 4.88 μg QuE/ml (*P* > 0.05), respectively, when 40, 70, and 95% ethanol were used. These indicated that 40, 70, and 95% ethanol solution exerted insignificant difference on the extraction of phenolics and flavonoids in PRA.

**TABLE 1:** Extraction rate, total phenolics, and total flavonoids content, and IC_50_ values for DPPH⋅ ABTS⋅^+^ scavenging ability, and α-glucosidase inhibition of different PRA extracts.

Ethanol concentration (%)	Extraction yield (%)	Phenolics (μg GAE/ml)	Flavonoids (μg QuE/ml)	DPPH⋅ (IC_50_, μg E./ml)	ABTS^+^ (IC_50_, μg E./ml)
E40	11.9	281.94 ± 3.36^a^	37.92 ± 5.64^a^	165.77 ± 14.11^a^	94.73 ± 2.06^b^
E70	18.1	276.82 ± 8.11^a^	36.67 ± 6.91^a^	151.31 ± 13.40^a^	119.89 ± 8.30^a^
E95	15.5	273.20 ± 9.55^a^	40.63 ± 4.88^a^	165.88 ± 4.68^a^	90.00 ± 7.02^b^
Quercetin				37.02 ± 0.24^b^	7.97 ± 0.61^c^

Values followed by different letter (a, b, and c) are significantly different (*P* < 0.05) as measured by Turkey’s *b* test using SPSS 22.0.

### 3.2. *In vitro* antioxidant abilities

Research had indicated that the activation of oxidative stress was closely associated with the pathological development of diabetic complications, and long-term hyperglycemia would in turn lead to the production of excessive ROS (8). Antioxidants can alleviate oxidative stress by clearing the over-produced free radicals or activating the endogenous antioxidant defense systems, thereby eliminating the progression of diabetes and related complications (19). Hence, it was meaningful to investigate the antioxidant activity of PRA extracts.

**FIGURE 1:**
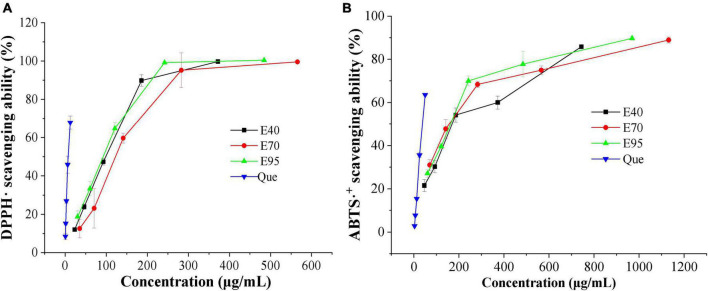
DPPH⋅ **(A)** and ABTS⋅^+^ **(B)** scavenging ability of 40% (E40), 70% (E70), and 95% (E95) ethanol extracts of PRA.

Two classic antioxidant models (DPPH⋅ and ABTS⋅^+^) were carried out to evaluate the antioxidant ability of PRA extracts. The DPPH⋅ and ABTS⋅^+^ scavenging capacity and corresponding IC_50_ values of PRA extracts and standard are shown in Figure 1 and Table 1, all ethanol aqueous extracts gave obvious radical scavenging ability in a dose-dependent manner, the IC_50_ values ranged from 151.31 to 165.88 μg E./ml for DPPH⋅ scavenging ability, and ranged from 90.00 to 119.89 μg E./ml for ABTS⋅^+^ scavenging ability, suggesting certain antioxidant potency. Currently, a variety of tannins, monoterpenoid, and flavonoids, especially for tannins have been identified in PRA, which might contribute to its antioxidant activity (20). But the activity was all lower than that of positive control quercetin. In addition, no significant difference was observed among the DPPH⋅ scavenging ability of extracts prepared with 40, 70, and 95% ethanol (*P* > 0.05). The 40 and 95% ethanol extracts exhibited stronger ABTS⋅^+^ scavenging ability than 70% ethanol extract, and insignificant difference was observed between E40 and E95 (*P* > 0.05).

### 3.3. Alleviation on H_2_O_2_-induced HepG2 oxidative stress

#### 3.3.1. Effect of PRA extracts on cell viability

**FIGURE 2:**
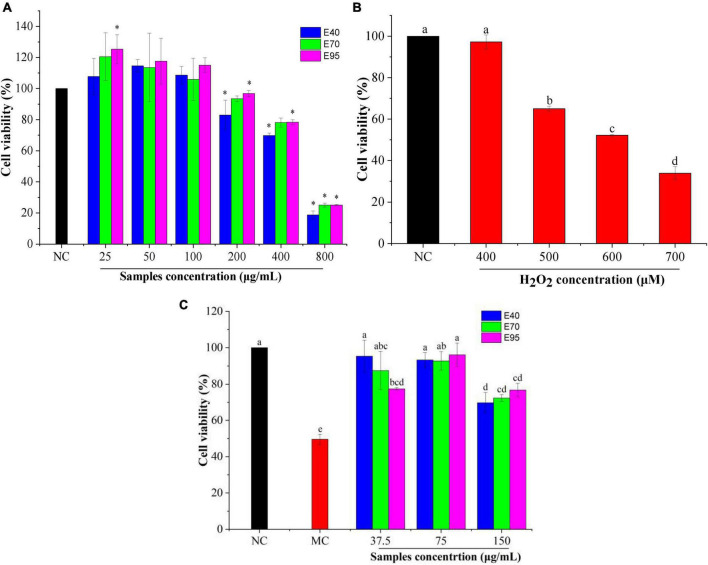
The viability of HepG2 cells treated with PRA extracts **(A)** and H_2_O_2_ **(B)**, effect of PRA extracts on cells viability of HepG2 cells induced by H_2_O_2_ **(C)**. Different letters (a, b, c, etc.) in each column indicate significant difference among the data (*P* < 0.05). *P < 0.05.

It is necessary to evaluate the toxic concentration of PRA extracts on HepG2 cells before determining its oxidative protective effect. Cell viability is often used as an indicator of cytotoxicity, and it was generally accepted that when the survival rate of cells exceeds 80% upon treated with a reagent, suggesting a non-cytotoxic effect (21). As shown in Figure 2A, exposure HepG2 cells to 25, 50, and 100 μg/ml of PRA extracts did not reduce the cell viability. Oppositely, induction with 25 μg/ml of E70 and E95 enhanced the viability. The viability of HepG2 cells remained above 80% when 200 μg/ml of E40, E70, or E95 was applied. While, the cell viability significantly decreased to 69.84 ∼ 78.41% when the concentration of PRA extracts arrived at 400 μg/ml, and dramatically to 18.79 ∼ 25.08% at 800 μg/ml, indicating severe HepG2 cells damage. Therefore, PRA extracts showed almost no cytotoxicity on HepG2 cells when the concentration was lower than 200 μg/ml, 37.5, 75.0, and 150 μg/ml of PRA extracts were thus selected for subsequent oxidative protection assays.

#### 3.3.2. PRA extracts enhanced the viability of H_2_O_2_-induced HepG2 cells

H_2_O_2_, as a considerably active oxygen molecule with relatively stable properties, is one of the main factors causing excessive oxidative stress, which is widely used to establish cell oxidative damage model (16, 22). In this research, 400 ∼ 700 μM of H_2_O_2_ were used to induce HepG2 cells for 6 h to establish the oxidative stress model. As shown in Figure 2B, compared with the untreated group, the cell viability declined in a concentration-dependent manner with the increasing of H_2_O_2_ concentration. The cell viability of HepG2 cells was reduced to 52.2% when induced with 600 μM H_2_O_2_ for 6 h (*P* < 0.01). Therefore, the concentration of 600 μM was selected to induce oxidative stress for subsequent experiment.

The protection effect of PRA extracts against oxidative damage were determined with the H_2_O_2_-induced HepG2 cells oxidative stress model. As shown in Figure 2C, the cell viability of model group (induced by H_2_O_2_) significantly decreased (*P* < 0.01) compared to the normal group (without H_2_O_2_ and extracts treatment), and reached to 49.56%, indicating the successful establishment of the oxidative damage model. It was greatly restored to 69.79 ∼ 96.16% when the cells were pre-treated with 37.5 ∼ 150 μg/ml of PRA extracts for 24 h prior to H_2_O_2_ incubation (*P* < 0.01). The highest viability was found on the cells pre-treated with 37.5 μg/ml of E40 or 75 μg/ml of E70 or E95, with the values enhanced by 1.87 ∼ 1.94-folds, indicating good alleviation of PRA extracts on cell oxidative damage. While, when the concentration was at 37.5 μg/ml, E40 and E70 treatment showed higher cell viability than E95, but no significant difference (*P* > 0.05) was observed among E40, E70, and E95 when the treatment concentration was at 75 or 150 μg/ml. The results disclosed that PRA extracts could significantly decrease the oxidative stress of HepG2 cells induced by H_2_O_2_, 40, 70, and 95% ethanol extracts showed similar oxidative protection, and the activity will be weaker when the induction concentration over 150 μg/ml.

#### 3.3.3. Effects on the levels of MDA and antioxidant enzymes

SOD, CAT, and GSH-Px are crucial endogenous antioxidant enzymes that provide the first line of defense against damage mediated by oxidative stress. The SOD responsible to catalyze O_2_^–^ to H_2_O_2_, O_2_ and less reactive H_2_O_2_, CAT can directly convert H_2_O_2_ into H_2_O and O_2_, GPXs are a large family of diverse isozymes that use glutathione to reduce H_2_O_2_ (21, 23). Intracellular MDA is a typical degradation product of lipid peroxidation in biological membranes, it can aggravate membrane damage and is often regarded as a biomarker of oxidative stress (22). Therefore, changes in the level of antioxidant enzymes (SOD, CAT, and GSH-Px) and MDA are regarded as pivotal indicator of antioxidant ability evaluation.

**FIGURE 3:**
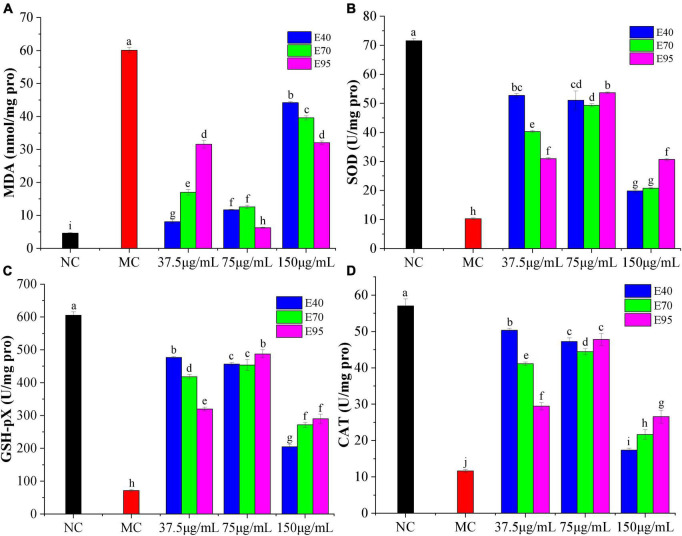
Effects of different concentrations of PRA extracts on the levels of MDA **(A)**, SOD **(B)**, GSH-Px **(C)**, and CAT **(D)** in H_2_O_2_-induced HepG2 cells. The different letters (a, b, c, etc.) upper each column indicate that there are significant differences among the data (*P* < 0.05).

Previous radical experiments showed that PRA could effectively scavenge free radicals *in vitro*, which meant that it might alleviate oxidative damage in HepG2 cells. As indicated in Figure 3A, the MDA level of HepG2 cells increased to 60.11 nmol/mg protein (*P* < 0.01) after being incubated with H_2_O_2_ for 6 h, indicating the occurrence of lipid peroxidation caused by H_2_O_2_-induced oxidative stress (24). Addition of PRA extracts could apparently prevent the release of MDA as compared to the model group (*P* < 0.01), the MDA levels were markedly reduced to 6.28 ∼ 12.6 nmol/mg protein when pre-treated with 75 μg/ml PRA extracts for 24 h, the best suppression was detected on 75 μg/ml of E95 (6.28 nmol/mg protein), followed by 37.5 μg/ml of E40 (8.08 nmol/mg protein). The suppression on MDA production was weaken when the pre-treatment concentration of PRA extracts reached 150 μg/ml, but the MDA levels were still much lower than that of model group.

As shown in Figures 3B–D, SOD, GSH-Px, and CAT levels decreased significantly in H_2_O_2_ induced HepG2 cells, the enzyme activity was reduced from 71.56, 605.41, and 57.03 U/mg protein in normal group to 10.28, 71.10, and 11.63 U/mg protein (*P* < 0.05), respectively, suggesting the generation of oxidative stress. Pre-treatment with 37.5 ∼ 150 μg/ml of PRA extracts for 24 h significantly restored the antioxidant enzyme activity, especially for extracts at the concentration of 75 μg/ml. The highest SOD, GSH-Px, and CAT level was individually found on the HepG2 cells pre-treated with 75 μg/ml of E95, 37.5 μg/ml of E40 and 75 μg/ml of E95 (*P* > 0.01), and 37.5 μg/ml of E40, the enzyme level was improved by 5.2, 6.7 ∼ 6.8, and 4.3-folds, respectively, as compared with the model group. Consistent with the results of cell viability, at a low pre-treatment concentration (37.5 μg/ml), E40 exhibited much better mitigation effect than E70 and E95 on H_2_O_2_-induced reduction on SOD, GSH-Px, and CAT levels. The ability did not vary greatly at middle treatment concentration, while, at high concentration of 150 μg/ml, the SOD, CAT, and GSH-Px levels were the lowest, indicating the presence of certain cytotoxicity, but they were still significantly higher than that of model group (*P* < 0.05). Therefore, it can be concluded that PRA extracts showed good protection on HepG2 cells against H_2_O_2_ induced oxidative stress through decreasing MDA formation and improving the levels of endogenous antioxidant enzymes SOD, CAT, and GSH-Px.

### 3.4. Inhibition on the activity of α-glucosidase and BSA glycosylation

#### 3.4.1. Inhibition on the activity of α-glucosidase

**FIGURE 4:**
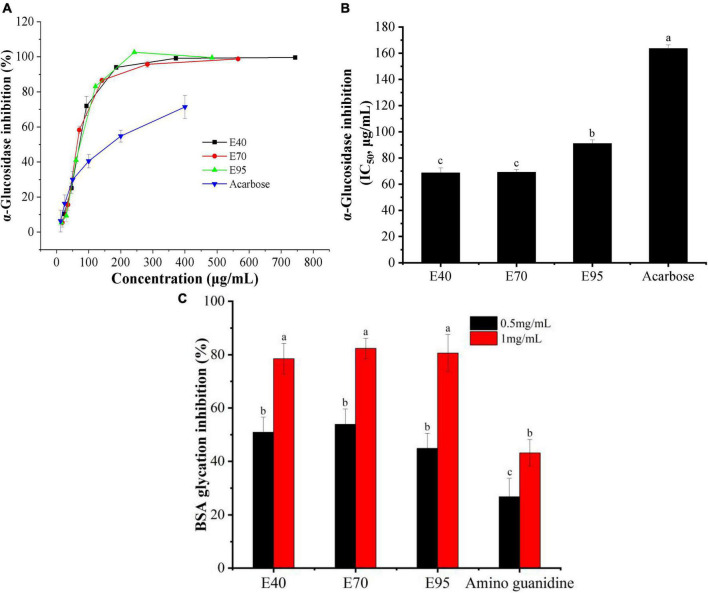
The α-glucosidase inhibition **(A)**, IC_50_ values for inhibiting α-glucosidase **(B)**, and anti-glycative ability **(C)** of PRA extracts. Different letters (a, b, c, etc.) upper each column indicate significant differences among the data (*P* < 0.05).

α-Glucosidase is a critical carbohydrate hydrolase secreted by intestinal epithelium, and responsible for the degradation of disaccharides, trisaccharides, and oligosaccharides into absorbable monosaccharides. Inhibiting the activity of α-glucosidase has been regarded as one of the important methods to control diabetes and diabetic complications (25). Hence, α-glucosidase inhibitory experiment was carried out to appraise the hyperglycemic activity of PRA extracts. Results were presented in Figures 4A, B. Obviously, all ethanol extracts displayed good α-glucosidase inhibitory activity, the percentage inhibition all over 85% when the concentration of PRA extracts arrived at 200 μg E./ml. Moreover, the α-glucosidase inhibition of E40, E70, and E95 all much higher than that of positive control acarbose, with the IC_50_ value of 68.58, 69.01, 91.07, and 163.56 μg/ml, respectively. The strongest inhibitory activity was found on E40 and E70 (*P* > 0.05), which was about 2.4 times of that of acarbose. Which is consistent with the results of Sun et al. (26), who found that ethanol extract of PRA can effectively inhibit α-glucosidase activity.

#### 3.4.2. Suppression on BSA glycosylation

Advanced glycation end products (AGE_*S*_) are a class of heterogeneous compounds spontaneously formed during the advanced stage of protein glycosylation. The accumulation of AGEs in body tissues will increase the oxidative stress and contribute to the pathology of various diabetic complications, such as retinopathy, neuropathy, and cardiovascular complications (27). Inhibiting protein glycosylation and AGEs accumulation have been considered as one of effective approach to alleviate diabetic complications. In this research, the inhibition of PRA extracts on protein glycosylation was evaluated with BSA-fructose model. As indicated in Figure 4C, the E40, E70, and E95 all exhibited stronger suppression on BSA glycosylation than standard amino guanidine, and displayed an obvious does-dependent relationship. While, no significant difference was found among E40, E70, and E95 at tested concentrations (*P* > 0.05). At the concentration of 1.0 mg/ml, the suppression ratio on AGEs formation of E40, E70, and E95 reached to 78.51, 82.40, and 80.63%, respectively, which was 1.82 ∼ 1.91 times of that of amino guanidine, suggesting the potential of PRA extracts in preventing diabetic complications.

### 3.5. Hypoglycemic activity on diabetic zebrafish model

#### 3.5.1. Hypoglycemic effect

During decades, zebrafish (*Danio rerio*) has been one of the favorite and validated model organism in screening of drug against metabolic diseases due to the advantage of high human genetic homology, easier visualization of tissues and organs, short drugs induction time, allowing the use of small amount of compound, etc. (28). In case of glucose metabolism, the biological mechanisms of zebrafish to regulate glucose homeostasis are very similar to those of humans, feeding on high glucose solution persistently can induce hyperglycemic symptoms and impair glucose metabolism (29). Therefore, hyperglycemic zebrafish model was developed in this research to evaluate the *in vivo* hypoglycemic activity of PRA extract.

According to the results displayed in Table 1 and Figures 1–4, the E70 exhibited the highest yield, good radical scavenging ability, excellent prevention on oxidative stress, promising α-glucosidase inhibition and anti-glycation. It was thus chosen as the representative sample for *in vivo* hypoglycemic activity evaluation. The toxicological concentration of E70 was analyzed with 5 dpf zebrafish reared in dechlorinated tap water containing 62.5 ∼ 2,000 μg/ml of E70 at 25°C. After 2 days (7.5 h/day) of treatment, no dead zebrafish was detected, suggesting the feasibility to use the concentration below 2.0 mg/ml in subsequent experiments.

**FIGURE 5:**
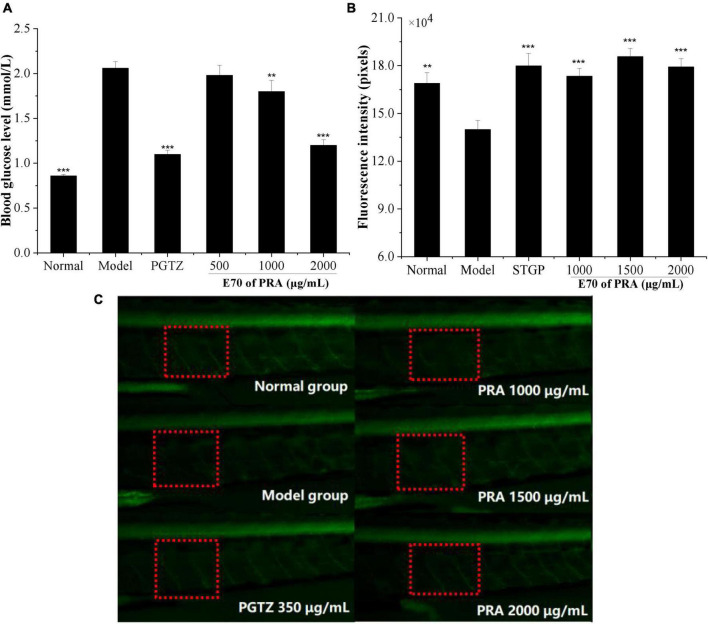
Blood glucose of zebrafish treated with E70 PRA extracts **(A)**, the fluorescence intensity of peripheral motor nerve of transgenic zebrafish **(B)**, and fluorescence diagram of peripheral motor nerve of zebrafish **(C)**. Compared with the model control group, **P < 0.01, ***P < 0.001. The red frame was the peripheral motor nerve, and the green fluorescence was the zebrafish motor neuron.

Effect of 0.5, 1.0, and 2.0 mg/ml of E70 on the blood glucose level of hyperglycemic zebrafish model induced by high-sugar and high-fat diet are shown in Figure 5. The blood glucose level of zebrafish in model group increased significantly from 0.86 mmol/L in normal group to 2.06 mmol/L (*P* < 0.001), which indicated that the model was successfully established. But it was reduced to 1.98, 1.80, and 1.20 mmol/L, respectively, upon treatment with 0.5, 1.0, and 2.0 mg/ml of E70, suggesting an obvious dose-dependent relationship. But no significant difference was observed between the glucose level of model group and 0.5 mg/ml of E70 treatment group (*P* > 0.05). When the concentration reached to 2.0 mg/ml, the blood glucose level decreased by 42% (*P* < 0.001), which was similar to that of 18 μg/ml of pioglitazone (47%), suggesting promising potential of high concentration of E70 in alleviating hyperglycemia. Studies have shown that paeony total glucosides could reduce blood glucose level by improving insulin sensitivity and lipid metabolism (30).

#### 3.5.2. Protective effect of peripheral motor nerve

Peripheral neuropathy is one of the typical chronic complications of diabetes, approximately 50% of adult patients suffering from diabetes developed various degrees of peripheral neuropathy in their lifetime (31). Dorsemans et al. (32) found that acute and chronic hyperglycemia impaired the regeneration and *de novo* formation of zebrafish neuronal cells, resulting to adverse effects on neurogenesis and brain healing. Alteration in the metabolic physiology associated with neurodegeneration was also found in the insulin resistance zebrafish larvae model by Razip et al. (33). To evaluate the prevention of E70 on diabetic complication, the protective effect of PRA on peripheral nerve was evaluated by detecting the fluorescence intensity of peripheral motor nerve of normal and hyperglycemic zebrafish. As indicated in Figures 5B, C, the fluorescence intensity of model group was 139,884 ± 5,656, which was much lower than that of normal group (168,917 ± 6,706), indicating the appearance of peripheral motor nerve damage in high glucose zebrafish model. Upon incubation with 1,000, 1,500, and 2,000 μg/ml of E70, the fluorescence intensities were increased to 173,347 ± 4,977, 185,685 ± 5,149, and 179,153 ± 5,248, respectively (*P* < 0.001), the value for these incubated with 350 μg/ml of standard sitagliptin was 179,871 ± 7,772. Above results suggested that the E70 of PRA could effectively mitigate peripheral motor nerve damage of hyperglycemic zebrafish. Huang et al. (34) showed that PRA extract might be a potential nerve growth promoting factor and could promote the growth of damaged peripheral nerves through *in vivo* and *in vitro* experiments.

### 3.6. Identification of phytochemical profiling

**FIGURE 6:**
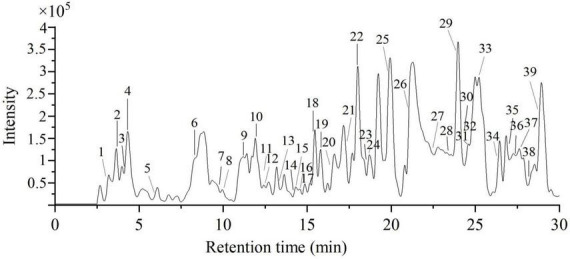
The total ion chromatography of 70% ethanol extract of PRA under negative mode.

In this work, the phytochemical constituents of E70 were further evaluated by HPLC-QTOF-MS/MS due to its excellent antioxidant and anti-diabetic activities. The composition was tentatively identified based on the molecular weight, fragment ions, retention time and formula. The total ion chromatogram and MS/MS information of E70 extract was respectively displayed in Figure 6 and Table 2. With the help of database and relevant literatures, a total of 35 compounds were identified in E70, which consisted of 5 organic acids, 4 phenolic acids, 12 tannins, 3 flavonoids, 8 terpenoids, and 3 other compounds.

**TABLE 2:** Identified and tentatively identified compounds in the 70% ethanol extract of PRA by HPLC-QTOF-MS/MS under negative ion mode.

No.	RT (min)	Formula	Found at m/z	Expected at m/z	Error (ppm)	MS/MS	Proposed compounds
**Organic acids**							
2	3.73	C_6_H_12_O_7_	195.0528	195.0510	−2.5	177.0429, 159.0250, 129.0266	Gluconic acid
3	4.15	C_9_H_14_O_8_	249.0629	249.0616	4.6	249.0638, 161.0445, 87.0087	Trimethylhydroxycitric acid
6	8.45	C_6_H_8_O_7_	191.0206	191.0197	4.5	191.0190, 173.0106, 111.0082, 87.0084	Citric acid
7	9.76	C_4_H_6_O_4_	117.0197	117.0193	−5.2	99.9266, 73.0330	Succinic acid
28	23.53	C_17_H_28_O_9_	375.1662	375.1661	6.6	161.0442	4-Hexosylmethyl-5-oxo-2-pentyltetrahydro-3 -furancarboxylic acid
**Phenolic acids**							
8	9.88	C_14_H_16_O_10_	343.0676	343.0671	1	343.0655, 191.0544, 169.0134	Galloylquinic acid
11	12.15	C_7_H_6_O_5_	169.0141	169.0143	1.6	169.0211, 125.0320, 107.0124, 97.0257	Gallic acid
12	12.52	C_14_H_18_O_9_	329.0881	329.0878	3.1	167.0371, 152.0123, 123.0444	Vanilloyl glucose
13	13.38	C_13_H_16_O_9_	315.0736	315.0722	6.7	153.0213	Protocatechuic acid hexoside
**Tannin**							
9	11.31	C_13_H_16_O_10_	331.0686	331.0671	2.5	331.0660, 271.0498, 211.0251, 169.0140	Monogalloyl-hexoside
10	11.92	C_19_H_26_O_15_	493.1216	493.1199	3.4	493.1275, 331.0703, 313.0605, 271.0486, 169.0159, 125.0268	6-*O*-galloylsucrose
14	14.18	C_27_H_22_O_18_	633.0769	633.0733	4.3	663.0839, 481.0870, 300.9992	Corilagin
17	15.04	C_13_H_24_O_19_	483.0791	483.0839	−9.2	313.0551, 169.0125	Digalloylglucopyranose
22	17.96	C_8_H_8_O_5_	183.0315	183.0299	8.6	183.0306, 124.0170, 78.0109	Methyl gallate
23	18.58	C_27_H_24_O_18_	635.0923	635.0890	4.7	635.0919, 465.0700, 313.0555, 169.0149	Trigalloylglucose
24	18.66	C_20_H_16_O_13_	463.0531	463.0518	2.9	301.0022, 299.9899	Ellagic acid-4-*O*-glucoside
29	23.94	C_9_H_10_O_5_	197.0474	197.0456	8.8	197.0493, 169.0183, 124.0203	Ethyl gallate
31	24.42	C_14_H_6_O_8_	301.0004	300.9990	3.3	301.0004, 283.9971, 229.0180	Ellagic acid
35	26.95	C_24_H_18_O_15_	545.0601	545.0573	5.1	469.0554, 393.0483, 169.0158	Dihydroxybenzoic acetate-digallate
36	27.56	C_15_H_12_O_9_	335.0423	335.0409	5.5	183.0347, 168.0088, 124.0208	Methyl digallate
38	28.20	C_24_H_18_O_15_	545.0606	545.0573	6.2	469.0553, 393.0469, 169.0158	Dihydroxybenzoic acetate-digallate isomer
**Flavonoids**							
16	14.49	C_21_H_24_O_11_	451.1255	451.1246	1.5	415.1313, 289.0734	(Epi)catechin glucopyranoside
21	17.28	C_15_H_14_O_6_	289.0742	289.0718	5.1	245.0885, 179.0586, 109.0332	(Epi)catechin
30	24.17	C_21_H_22_O_10_	433.1161	433.1140	6	313.0620, 271.0609, 151.0034	Naringenin-C-glucoside
**Terpenoids**							
19	15.76	C_23_H_28_O_12_	495.1520	495.1508	2.7	495.1567, 465.1456, 137.0270	Oxypaeoniflorin
20	16.66	C_23_H_28_O_11_	479.1570	479.1559	2.3	479.1661, 283.1701, 167.0345	Paeoniflorin
25	19.88	C_23_H_28_O_11_	479.1564	479.1559	1	479.1661, 121.0343	Paeoniflorin isomer
26	21.02	C_23_H_28_O_11_	479.1562	479.1559	0.7	449.1475, 327.1099, 165.0554, 121.0307	Paeoniflorin isomer
27	22.44	C_23_H_28_O_12_	495.1527	495.1508	3.9	495.1575, 465.1467, 137.0293	Oxypaeoniflorin isomer
32	24.74	C_30_H_32_O_15_	631.1695	631.1668	2.7	465.1406, 313.0559, 169.0155	Galloylpaeoniflorin
34	26.34	C_30_H_32_O_15_	631.1686	631.1668	2.7	631.1686, 313.0574, 169.0165	Galloylpaeoniflorin isomer
39	28.79	C_23_H_28_O_11_	479.1566	479.1559	1.4	479.1560, 283.0819, 121.0303	Paeoniflorin isomer
**Others**							
4	4.32	C_12_H_22_O_11_	341.1089	341.1089	0.1	179.0572	Sucrose
15	14.32	C_21_H_32_O_10_	433.1937	443.1923	1.9	101.0242	Penstemide
37	27.57	C_16_H_26_O_8_	345.1571	345.1555	4.3	345.1601, 179.0558, 165.0959, 59.0163	Picrocrocinic acid

#### 3.6.1. Organic acid

Peak 2 was identified as gluconic acid, the MS/MS ion at 159.0250 indicated the loss of two H_2_O. Peak 6 with fragment ion at 111.0082 ([M-H-CO_2_-2H_2_O]^–^) was assigned as citric acid. Peak 3 was proposed as trimethylhydroxycitric acid, the MS/MS ion at 161.0445 was produced by loss of two -CO_2_. Peak 7 was suggested as succinic acid resulted from the characteristic fragment ion at 73.0330 ([M-H-CO_2_]^–^). Peak 28 was proposed as 4-hexosylmethyl-5-oxo-2-pentyltetrahydro-3-furancarboxylic acid by matching the MS/MS ion with MassBank database (35).

#### 3.6.2. Phenolic acids

Peak 11 was identified as gallic acid by comparing with standard due to the characteristic fragment ion at 169.0211 ([gallic acid-H]^–^). Peak 8 was proposed as galloylquinic acid based on the MS/MS ions at 191.0544 and 169.0134 (36). Peak 13 with MS/MS ion at 153.0213 [M-162-H]^–^ implied the presence of protocatechuic acid and loss of hexoside, and was suggested as protocatechuic acid hexoside (37).

#### 3.6.3. Tannins

Peaks 9, 10, 17, and 23 were identified as gallic acid derivatives due to the diagnostic MS/MS ion at 169. Based on the MS/MS information, peaks 9 and 10 existed a hexose and sucrose moieties, and were characterized as monogalloyl-hexoside and galloylsucrose, respectively. Peaks 17 and 23 were suggested as digalloylglucopyranose and trigalloylglucose, respectively, the MS/MS ions at 313 and 465 indicated the presence of two and three galloyl groups. Peak 14 was proposed as corilagin, the MS/MS ion at 300.9992 was generated by the loss of a gallic acid and a glucose residue (18). Peaks 22 and 29 with characteristic fragment ion at 124.02 (C_6_H_4_O_3_) demonstrated the loss of trihydroxyphenyl group, and were individually proposed as methyl gallate and ethyl gallate according to literature (38). Peak 31 with MS/MS ions at 301.00, 283.99, and 229.02 was identified as ellagic acid by comparing with literature (39). Peak 24 ([M-H]^–^, 463.0531, C_20_H_16_O_13_) was suggested as ellagic acid-4-*O*-glucoside, and was the glucoside substituted compound of peak 31. Peaks 35 and 38 showed the same molecular formula (C_24_H_18_O_15_) were assigned as dihydroxybenzoic acetate-digallate isomers, the MS/MS ions at 469.0553 [M-H-72]^–^ and 393.0469 [M-H-152]^–^ corresponded to the loss of hydroxyacetyl group and galloyl groups (18). Peak 36 was galloyl substituted compound of peak 22, and was identified as methyl digallate.

#### 3.6.4. Flavonoids

Three flavonoids were detected in E70. Peak 16 was suggested as (epi)catechin glucopyranoside, the MS/MS ion at 289.0734 represented the existence of (epi)catechin (40). Peak 21 with MS/MS ions at 245.0885, 179.0586, and 109.0332 accounted for the characterization of (epi)catechin. Peak 30 with MS/MS ion at 271.0609 displayed the loss of glucoside, the characteristic fragment at 151.0034 was produced by naringenin, which was identified as naringenin-C-glucoside (41).

#### 3.6.5. Terpenoids

Peaks 20, 25, 26, and 39 possessed the same MS ion at 479.1559, and was suggested as paeoniflorin isomers. The MS/MS ions at 283 and 121 indicated the loss of benzoic acid and glucose moieties (42). The molecular weight of peaks 19 and 27 was 16 Da higher than that of peak 20, and were tentatively identified as oxypaeoniflorin isomers. Similar, peaks 32 and 34 gave the same molecular formula (C_30_H_32_O_15_), the molecular weight was 152 Da higher than peak 20 due to the loss of a galloyl group, which were proposed as galloylpaeoniflorin isomers.

#### 3.6.6. Others

Peak 4 was characterized as sucrose through standard. Peak 15 with MS/MS ion at 101.0242 ([M-H-C_16_H_23_O_8_]^–^) was identified as penstemide (40). Peak 37 was assigned as picrocrocinic acid by matching the MS/MS ions with that reported by Zhu et al. (43).

## 4. Discussion

In recent years, the prevalence of diabetes, especially type 2 diabetes, has been increasing. Uncontrolled diabetes can lead to many complications (neuropathy, nephropathy, retinopathy, cardiovascular diseases, etc.), which is also the cause of its high mortality. Oxidative stress has always been considered as a key factor in the development and progress of diabetes and its related complications, many studies are devoted to the role of antioxidants in diabetes (44). It has been proved that plant extracts were excellent antioxidants and had potential applications in reducing oxidative damage of diabetes (45). PRA contained a variety of bio-active compositions, which was a natural product with high antioxidant and hypoglycemic potential (11, 20).

In our research, 70% ethanol exhibited the highest extract yield as compared with 40% ethanol and 95% ethanol. The E70 presented excellent free radical scavenging ability, anti-glycation ability, as well as the best α-glucosidase inhibition. In addition, the oxidative damage protection and *in vivo* hypoglycemic activity of PRA were evaluated by H_2_O_2_-induced HepG2 cells oxidative damage model and diabetic zebrafish model, respectively. Markedly, the cell survival rate of H_2_O_2–_induced HepG2 cells was increased when pre-treatment with PRA. The activities of SOD, CAT, and GSH-Px were obviously enhanced, and the level of MDA in cells was remarkably decreased. The results turned out that PRA extracts had good protection effect on HepG2 cells against damage induced by H_2_O_2_. UPLC-QTOF-MS/MS analysis showed that the main components of PRA were tannins (gallotannins, gallic acid, and its derivatives) and terpenoids (paeoniflorin and its derivatives), which were consistent with the found of Xiong et al. (18).

Tannins, especially for hydrolysable tannins, have been proved to show antioxidant, anti-glycation, and hypoglycemic activity by a multiple of researchers (46, 47). Yamini Kosuru et al. (48) summarized that gallic acids and their derivatives could improve the antioxidant capacity by increasing antioxidant enzymes (SOD, CAT, and GSH-Px) and decreasing lipid per-oxidant. Ding et al. (49) indicated that ellagic acid ameliorated oxidative stress by reducing ROS and MDA levels and increasing SOD activity via activating miR-223-mediated keap1-Nrf2 pathway in high glucose-induced HepG2 cells. In case of terpenoids, Parker et al. (20) summarized that paeoniflorin possessed the highest concentration in PRA, it was also found abundance in E70. Yuan et al. (50) reported that paeoniflorin alleviated oxidative stress by increasing SOD and CAT levels in H_2_O_2_-induced HepG2 cells.

Oxidative stress was highly associated with DM and its complications (6). The results indicated that E70 PRA extract significantly reduced the blood glucose level and alleviated peripheral motor nerve damage in diabetic zebrafish. Similarly, paeoniflorin was reported to be able to protected pancreatic β cells from streptozotocin-induced damage by inhibiting the p38 MAPK and JNK signaling pathways, and then maintained blood glucose level (51). Further, Laddha and Kulkarni (52) reported that hydrolysable tannins, such as gallic acid played a vital role in diabetic peripheral neuropathy because of its antioxidant and insulin secretion promoting effects.

Based on above results, tannins, and terpenoids had excellent antioxidant and hypoglycemic activities, the abundance gallotannins, gallic acid and its derivatives, and paeoniflorin and its derivatives contributed to the promising antioxidant, anti-glycation, and hypoglycemic activities of PRA extracts.

## 5. Conclusion

In summary, our research showed that 40, 70, and 95% showed insignificant difference on the extraction of phenolics and flavonoids from PRA, all ethanol aqueous extracts of PRA could effectively scavenge free radical, inhibit the activity of α-glucosidase and BSA glycosylation. Meanwhile, PRA extracts pre-treatment alleviated the H_2_O_2_-induced oxidative damage of HepG2 cells by increasing the activity of antioxidant enzymes SOD, CAT, and GSH-Px and reducing the production of MDA. Furthermore, E70 extract exhibited excellent hypoglycemic activity *in vivo*, the blood glucose level of diabetic zebrafish induced by high sugar and high fat was reduced by 42% when treatment with 2.0 mg/ml of E70, which was similar to that of 18 μg/ml of pioglitazone (47%). The peripheral motor nerve damage of hyperglycemic zebrafish was also obviously mitigated by 1.0 ∼ 2.0 mg/ml of E70. Totally, 35 compounds were identified or tentatively identified from E70 by HPLC-QTOF-MS/MS, terpenoids (paeoniflorin and its derivatives) and tannins (gallotannins, gallic acid and its derivatives) were the dominant antioxidant and hypoglycemic active constituents of E70. To sum up, these results indicated that PRA ethanol extracts may be a promising potential natural antioxidant and hypoglycemic resource to prevent the chronic degenerative disease caused by oxidative stress and prolonged high blood sugar.

## Data availability statement

The original contributions presented in this study are included in the article/supplementary material, further inquiries can be directed to the corresponding author.

## Ethics statement

This animal study was reviewed and approved by the Institutional Animal Care and Use Committee at Hunter Biotechnology, Inc., [approval number: IACUC-2020-2574-01, use license number: SYXK (zhe) 2022-0004].

## Author contributions

LZ: investigation, methodology, formal analysis, and writing—review and editing. MD: investigation, validation, formal analysis, and writing. S-YW: methodology, investigation, and original draft. QD: software, investigation, and statistical analysis. J-HL: investigation and validation. XX: supervision, methodology, and writing—review and editing. Y-HH: conceptualization, supervision, and methodology. Z-CT: funding acquisition and project administration. All authors contributed to the article and approved the submitted version.
